# Extracellular cAMP-Adenosine Pathway Signaling: A Potential Therapeutic Target in Chronic Inflammatory Airway Diseases

**DOI:** 10.3389/fimmu.2022.866097

**Published:** 2022-04-11

**Authors:** Enio Setsuo Arakaki Pacini, Naiara Ayako Satori, Edwin Kerry Jackson, Rosely Oliveira Godinho

**Affiliations:** ^1^Division of Cellular Pharmacology, Department of Pharmacology, Universidade Federal de São Paulo, São Paulo, Brazil; ^2^Department of Pharmacology and Chemical Biology, University of Pittsburgh School of Medicine, Pittsburgh, PA, United States

**Keywords:** adenosine, adenosine receptors (AR), cyclic AMP (cAMP), airway, chronic inflammatory diseases, inflammation, ecto-phosphodiesterase, ecto-5’nucleotidases

## Abstract

Adenosine is a purine nucleoside that, *via* activation of distinct G protein-coupled receptors, modulates inflammation and immune responses. Under pathological conditions and in response to inflammatory stimuli, extracellular ATP is released from damaged cells and is metabolized to extracellular adenosine. However, studies over the past 30 years provide strong evidence for another source of extracellular adenosine, namely the “cAMP-adenosine pathway.” The cAMP-adenosine pathway is a biochemical mechanism mediated by ATP-binding cassette transporters that facilitate cAMP efflux and by specific ectoenzymes that convert cAMP to AMP (ecto-PDEs) and AMP to adenosine (ecto-nucleotidases such as CD73). Importantly, the cAMP-adenosine pathway is operative in many cell types, including those of the airways. In airways, β_2_-adrenoceptor agonists, which are used as bronchodilators for treatment of asthma and chronic respiratory diseases, stimulate cAMP efflux and thus trigger the extracellular cAMP-adenosine pathway leading to increased concentrations of extracellular adenosine in airways. In the airways, extracellular adenosine exerts pro-inflammatory effects and induces bronchoconstriction in patients with asthma and chronic obstructive pulmonary diseases. These considerations lead to the hypothesis that the cAMP-adenosine pathway attenuates the efficacy of β_2_-adrenoceptor agonists. Indeed, our recent findings support this view. In this mini-review, we will highlight the potential role of the extracellular cAMP-adenosine pathway in chronic respiratory inflammatory disorders, and we will explore how extracellular cAMP could interfere with the regulatory effects of intracellular cAMP on airway smooth muscle and innate immune cell function. Finally, we will discuss therapeutic possibilities targeting the extracellular cAMP-adenosine pathway for treatment of these respiratory diseases.

## Introduction

Adenosine is an endogenous purine nucleoside that, *via* activation of specific adenosine receptors, modulates inflammation and immune responses (1–3). Adenosine receptors are seven transmembrane G-protein coupled receptors (GPCRs) (4, 5), and consist of a family of four adenosine receptor subtypes called A_1_, A_2A_, A_2B_ and A_3_. These four receptors are encoded by different genes and present high affinities for the α subunit (Gα) of heterotrimeric G-proteins that regulate adenylyl cyclase (AC) activity (6). A_1_ and A_3_ receptors are preferentially coupled to the inhibitory Gα (Gi/o) subunit that inhibits AC1, AC5 and AC6, decreasing 3’,5’-cAMP (from here forward referred to as simply cAMP) production, whereas A_2A_ and A_2B_ are strongly coupled to stimulatory Gα (Gs) subunits, which can activate all nine membrane-bound AC isoforms, increasing intracellular cAMP concentrations (7, 8) and triggering one or more cAMP-dependent intracellular signaling pathways (9). cAMP-dependent protein kinase (PKA) is by far the best studied effector of cAMP signaling and regulates many cellular processes including cell proliferation and differentiation, among others (10). By interacting with specific domains in the PKA regulatory subunits, cAMP releases the two catalytic PKA subunits to phosphorylate serine and/or threonine residues in target proteins (11). A_2_ receptor signaling may also be mediated by cAMP/EPAC (exchange protein directly activated by cAMP) pathways (12). Notably, adenosine receptors can also activate other signaling molecules *via* canonical GPCR pathways, such as phospholipase C β (PLC β) and Ca^2+^ *via* activation of A_1_, A_2A_ and A_3_ receptors (4) or by G protein-independent mechanisms mediated by β-arrestin (A_1_ and A_2_) (13).

Although cAMP is capable of diffusing throughout the cell, cAMP signaling occurs in microdomains or even nanodomains (14), which are at least in part created by intracellular phosphodiesterases (PDE), metallohydrolases involved in the degradation of cyclic nucleotides and distributed in 11 families (PDE 1–11). While members of PDE 4, 7 and 8 families selectively hydrolyze cAMP, PDE 1-3 and 10-11 are able to hydrolyze both cAMP and 3’,5’-cGMP (15). Intracellular compartmentalization of cAMP signaling also involves A kinase anchoring proteins (AKAPs), which bind to specific domains of PKA regulatory subunits and thereby guide PKA to different subcellular location (16). AKAPs also associate with PDEs, thus influencing the phosphorylation of target proteins and the termination of PKA activation.

## Adenosine and Inflammatory/Immune Responses

Several studies associate adenosine with a pro-inflammatory response (17), but many others have linked high concentrations of extracellular adenosine to a protective anti-inflammatory (18–20) and pro-resolving functions (2, 21). It seems that adenosine can limit the progression of inflammatory processes by reducing leukocyte infiltration and the production of inflammatory mediators, as well as by inducing apoptosis of inflammatory cells and promoting tissue repair; for review see (22). In general, these protective effects of adenosine have been associated with A_2A_ receptor activation, since they are drastically reduced in A_2A_-deficient mice, resulting in increased tissue damage and accumulation of pro-inflammatory cytokines (23, 24).

In fact, because adenosine binds with different affinities to its receptor subtypes (Ki: A_1_ = ~100 nM; A_2A_ = ~310 nM; A_3_ = ~290 nM and A_2B_ = ~15,000 nM) (4), its final effect will depend on both the receptor subtype expressed in the target cell and the extracellular concentration of adenosine. Thus, while at early stages of inflammation, neutrophil recruitment, phagocytosis and adhesion to the vascular endothelium are promoted by low concentrations of adenosine *via* activation of A_1_ and A_3_ receptors (25–27), during the healing phase, neutrophil recruitment is inhibited by high concentrations of adenosine *via* A_2A_ receptors (28).

By activating A_2A_ and A_2B_ receptors, adenosine inhibits inflammatory functions of neutrophils, reducing phagocytosis, degranulation, production of reactive oxygen species (ROS) (25, 29–32) and release of pro-inflammatory mediators (33–35). *Via* A_2A_ and A_2B_ receptors, adenosine also inhibits monocyte proliferation and differentiation into macrophages, reduces macrophage phagocytosis, attenuates the oxidative burst, and decreases the production of pro-inflammatory mediators such as TNF-α and IL-12 (36). Regarding lymphocytes, activation of A_2A_ receptors by adenosine reduces the production of inflammatory cytokines by T helper cells (37), and inhibits the development and activation of effector cells (38). Finally, adenosine also influences mast cells and eosinophils (17), important players in physiological innate immunity and allergic inflammatory diseases.

## Adenosine Signaling and the Regulation of Airway Inflammatory Diseases

Many of adenosine effects on airways involve modulation of intracellular cAMP concentrations on innate and adaptive immune cells (39). Although complex, in general, elevated intracellular cAMP concentrations mediate anti-inflammatory effects and immune suppression; for review see (40). By activating PKA, cAMP inhibits release of pro-inflammatory cytokines from dendritic cells (IL-12 and TNF-α) (41) and macrophages (TNF-α, MIP-1α, and LTB4) (42). cAMP also inhibits T cell chemotaxis (43) and antigen-stimulated B cell proliferation (44).

In the airways, the extracellular adenosine concentrations are relatively low under normal physiological conditions, ranging from 20 to 300 nM (45, 46). However, adenosine concentrations drastically increase in bronchoalveolar lavage fluid and exhaled breath condensate in patients with asthma, COPD and cystic fibrosis (18, 47–50), reaching micromolar to millimolar range. These high concentrations of adenosine are associated with bronchoconstriction and airway smooth muscle hyperresponsiveness (51–54), indicating the involvement of adenosine in the pathophysiology of pulmonary inflammatory diseases. Adenosine induces bronchoconstriction mainly through direct activation of A_1_ receptors expressed by smooth muscle cells (55). Other studies have associated adenosine-induced bronchoconstriction with release of inflammatory mediators, such as histamine and leukotrienes from mast cells; accordingly, adenosine-induced airway smooth muscle contraction is attenuated by H_1_‐histamine and CysLT_1_ leukotriene antagonists (56–58). Moreover, indirect bronchoconstriction induced by adenosine may also involve activation of A_1_ receptors on vagal afferent neurons. The evidence for this is that vagotomy and inhibitors of cholinergic pathways attenuate adenosine-induced bronchoconstriction (59).

The contribution of adenosine to airway inflammation is multifaceted and involves different receptors and intracellular signaling pathways in dendritic cells, monocytes, macrophages, neutrophils, bronchial smooth muscle and epithelial cells. Pro-inflammatory or anti-inflammatory airway responses are largely dependent on adenosine concentration and disease state over time. Particularly during acute airway inflammation, adenosine induces pro-resolving and tissue-protective actions, while in chronic airway inflammation it promotes deleterious effects such as tissue damage, fibrosis and release of pro-inflammatory cytokines (60, 61). Activation of A_2B_ receptors induces release of pro-inflammatory cytokines such as IL-6 from bronchial smooth muscle cells (62) and lung fibroblasts (63). Adenosine also affects the function of airway epithelial cells (e.g., ciliated cells, mucous cells, secretory cells, and basal cells) (64). While activation of A_1_ receptors increases production and secretion of mucus (65, 66) and modulates Cl^-^ transport in normal and cystic fibrosis human airway epithelial cells (67, 68), activation of A_2A_ receptors elicits a robust release of pro-inflammatory IL-6, IL-8 and TNF-α from airway epithelia (60, 69, 70). Adenosine also potentiates the recruitment of eosinophils and mast cells (53, 71) and induces airway inflammation by stimulating mast cell degranulation and release of pro-inflammatory mediators such as of IL-5, IL-13 as well as IL-4 (72, 73).

Investigations using adenosine deaminase (ADA)-deficient mice have also provided compelling evidence that accumulation of adenosine in the lung can lead to the development and exacerbation of chronic lung disease (74). ADA-deficient mice exhibit severe pulmonary inflammation and pathological features resembling those seen in asthma and chronic obstructive pulmonary diseases. These features include accumulation of macrophages and eosinophils, enlargement of alveolar spaces, increased production of mucus, higher concentrations of pro-inflammatory cytokines such as IL-5 and IL-13 and increased mast cell degranulation (75–77). Interestingly, all of these changes can be reversed by lowering the adenosine concentrations or by adenosine receptor antagonists (75, 78).

## Extracellular cAMP as a Source of Adenosine

The canonical pathway of adenosine formation involves sequential dephosphorylation of ATP into ADP and AMP mediated by intra- or extracellular nucleoside triphosphate diphosphohydrolases (NTPDases), alkaline phosphatases (APs), nucleotide pyrophosphatase/phosphodiesterases (NPPs) and 5’-nucleotidases (5-NTs) (79). A second pathway requires intracellular hydrolysis of S-adenosyl-homocysteine by the enzyme S-adenosyl-L-homocysteine hydrolase (80), and a third mechanism is mediated by the metabolism of extracellular NAD^+^ to adenosine by a CD38/CD203a/CD73 ectoenzymatic pathway (81).

**Figure 1:**
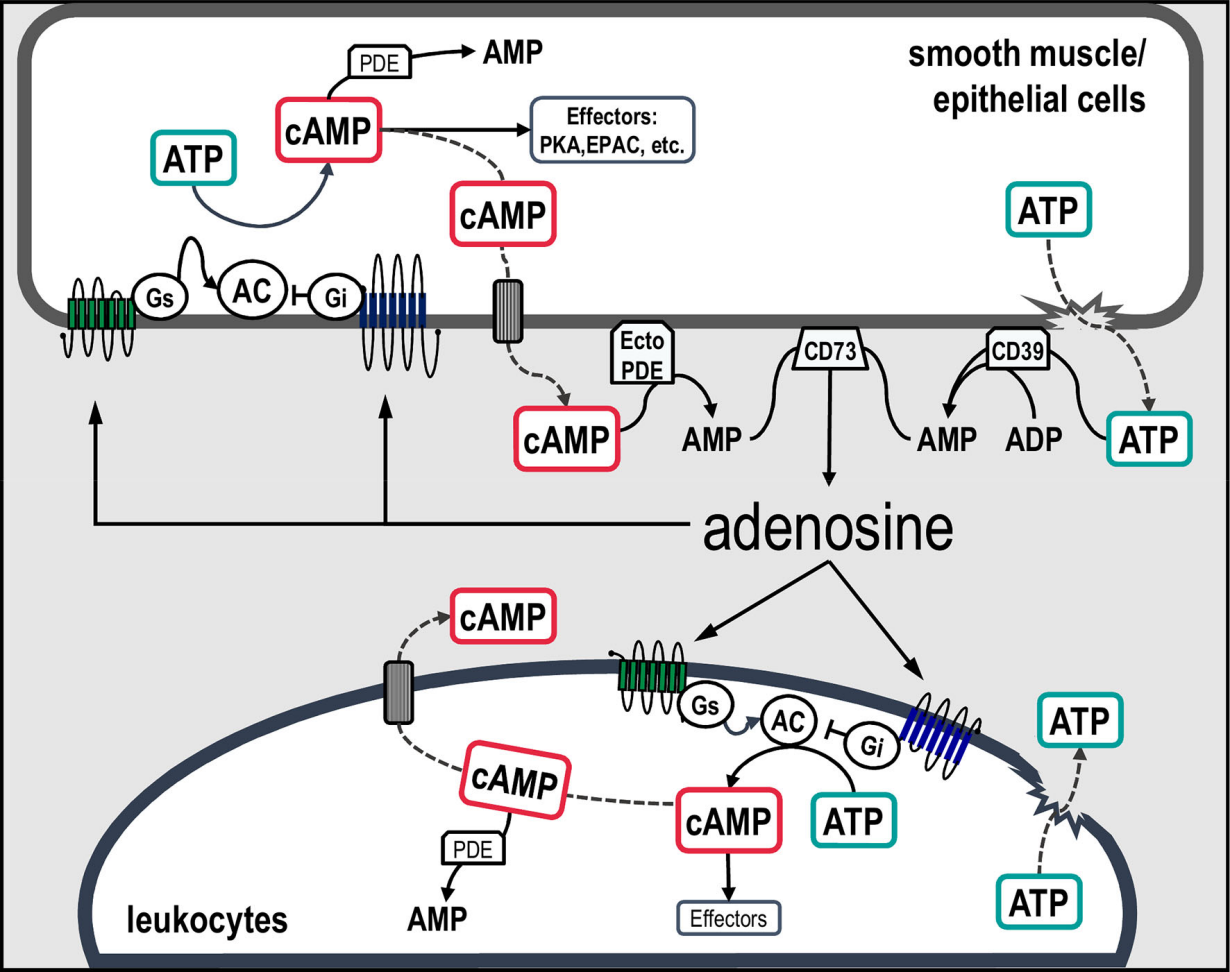
Schematic representation of canonical and non-canonical pathways involved in extracellular adenosine formation in airway cells. Classically, extracellular adenosine is generated mainly *via* the dephosphorylation of ATP, ADP and AMP by the action of a series of ecto-enzymes. An alternative source of adenosine is the second messenger cAMP. In this scenario, intracellular cAMP synthetized by adenylyl cyclases can be transported to the extracellular compartment and sequentially metabolized into AMP and adenosine by ecto-PDE and CD73, respectively. Increased concentrations of adenosine activate adenosine receptors coupled to Gs or Gi proteins, which in turn regulates the activity of adenylyl cyclases leading to an increase or decrease in cAMP concentrations and stimulation of downstream effectors. AC, adenylyl cyclases; Gs, stimulatory G protein; Gi, inhibitory G protein; PDE, phosphodiesterase; PKA, cAMP-dependent protein kinase; EPAC, exchange protein directly activated by cAMP; CD39, NTPDase-1; CD73, ecto-5’-nucleotidases and Ecto-PDE, ecto-phosphodiesterase.

In addition to the aforementioned pathways of adenosine production, cAMP is now widely recognized as an important source of extracellular adenosine (82). This biochemical route was originally described in the kidney by Jackson in the early 1990s (83) who named this mechanism the “extracellular cAMP-adenosine pathway” (84, 85). This pathway involves the sequential metabolism of extracellular cAMP to 5’-AMP and adenosine by ecto-phosphodiesterases (ecto-PDE) and ecto-5’-nucleotidases (CD73), respectively (86–88) (**Figure 1**).

Ecto-PDE activity has been described in many tissues, including vascular smooth muscle, kidney, intestine, skeletal muscle and airway, and in bovine seminal plasma and epididymal fluid (85, 88–92). Few research groups, however, have attempted to characterized ecto-PDE. Pharmacological studies using selective inhibitors of the intracellular cAMP-specific PDE isoforms demonstrate that ecto-PDEs obtained from the kidney, seminal plasma and vesical fluid present characteristics similar to those of the intracellular PDE8 and PDE10 families (89, 91).

The canonical pathway of extracellular adenosine production requires extracellular ATP as a precursor. In this regard, ATP can be specifically released *via* vesicular exocytosis and membrane pores upon cell activation or from damaged cells after transient or persistent inflammatory stimuli (93, 94). In contrast, the extracellular cAMP-adenosine pathway requires transport of intracellular cAMP to the extracellular compartment, a process that is mediated by members of ATP-binding cassette (ABC) transporters, subfamily C (ABCC). Due to their ability to extrude various chemotherapeutic agents from tumor cells, ABCC transporters are also referred to as multidrug resistance proteins (MRPs or MDRs). This family of transporters consist of nine members (ABCC1-ABCC9) that use ATP hydrolysis to mediate the efflux of multiple cellular substrates (95). Currently there are four well-defined ABCC proteins (ABCC1/MRP1, ABCC4/MRP4, ABCC5/MRP5 and ABCC11/MRP8) that can transport cAMP out of the cell, with specific kinetic parameters (96, 97); all of these cAMP transporters are expressed in human airway smooth muscle and epithelial cells (98–102). Recent studies analyzing GSE datasets from GEO (Gene Expression Omnibus) show that in airway epithelial cells the rank order of ABCC gene expression is ABCC5 >ABCC1 >ABCC4 >ABCC11 (101), whereas in the airway smooth muscle cells it is ABCC1 >ABCC4 >ABCC5 >ABCC11 (102).

## Extracellular cAMP-Adenosine Pathway in Airways and Potential Role in Chronic Inflammatory Diseases

Because of the importance of intracellular cAMP in the physiological modulation of airway smooth muscle tone and as a signaling molecule that mediates the action of bronchodilator agents (e.g., β_2_-adrenoceptor agonists used for rescue treatment of asthmatic patients suffering acute bronchoconstriction), we have focused our studies on the potential role of extracellular cAMP in the regulation of airway smooth muscle tone and as a source of adenosine. Using isolated rat trachea, we have shown that fenoterol (short-acting) and formoterol (long acting) β_2_-adrenoceptor agonists induce a time-dependent increase in extracellular cAMP concentrations (54). Interestingly, extracellular cAMP triggers a concentration-dependent contraction of airway smooth muscle that is mimicked by adenosine and increased by drugs that inhibit adenosine degradation (EHNA) and uptake (uridine), indicating that the contracting effects of extracellular cAMP depends on extracellular adenosine formation (54). Indeed, treatment of trachea segments with the adenosine receptor antagonist GCS-15943 increases the potency of fenoterol with regard to inducing smooth muscle relaxation, indicating that the extracellular cAMP-adenosine pathway compromises, *via* activation of adenosine receptors, the efficacy of β_2_-adrenoceptor agonists as bronchodilators. Further supporting this idea, using ultraperformance liquid chromatography–tandem mass spectrometry (LC-MS/MS), we found that incubation of isolated rat tracheas with cell impermeable cAMP leads to increases in extracellular concentrations of 5’-AMP, adenosine and inosine (92). The involvement of ecto-phosphodiesterases and ecto-5’-nucleotidase/CD73 in the extracellular metabolism of cAMP is demonstrated by the fact the selective inhibitors of these ecto-enzymes (DPSPX and AMP-CP, respectively) significantly reduce adenosine concentrations (92). Consistent with these observations, studies by Huff *et al.* (103) demonstrate that cAMP efflux from human airway epithelial cells occurs and is mediated by ABBC4/MRP4 transporters. More recently, Cao and coworkers (102) showed that stimulation of β_2_-adrenoceptors or AC with formoterol or forskolin, respectively, also promotes extrusion of cAMP from human airway smooth muscle cells in culture. cAMP efflux is markedly reduced by pharmacological inhibition or downregulation of ABCC1 transporters with siRNA, resulting in potentiation of β-agonist induced airway smooth muscle relaxation (102). Collectively, these findings reveal the existence of a functional extracellular cAMP-adenosine pathway in airways.

Increase in plasma concentration of cAMP is observed in physiological and pathological conditions such as during spontaneous or PGE-induced labor (104), insulin-induced hypoglycemia (105) in malignant hyperpyrexia susceptible individuals (106) or following traumatic injury. In fact, according to Cock et al. (107),, there is a positive correlation between the plasma concentrations of cAMP and neutrophil count following injury, which supports the idea that neutrophil mobilization can be activated by extracellular cAMP. Also, cAMP extrusion from neutrophils is stimulated by PGE_1_, isoproterenol, or forskolin (108), indicating that many cells are able to release cAMP. Regarding the airways, based on the extracellular fluid volume of rat trachea (~1 ml/g of dry tissue weight) (109), we have estimated that after β_2_-adrenoceptors stimulation, extracellular cAMP can reach micromolar concentrations (54), which are those required to induce smooth muscle contraction.

Studies addressing the expression of transporters and ecto-enzymes responsible for efflux and extracellular degradation of cAMP in the airway cells support a potential role for the extracellular cAMP-adenosine pathway in the pathophysiology of different respiratory diseases. Airway epithelial cells from tobacco smokers and asthmatic patients have increased expression of ABCC1, in comparison with those of healthy individuals (101), which is consistent with the increased concentrations of serum cAMP in asthmatic patients (102). With regard to ecto-enzymes, several studies using human tissues or animal models of respiratory diseases reveal increased expression and enzymatic activity of ecto-5’-nucleotidase/CD73, which could explain the higher level of extracellular adenosine associated with mechanical ventilation-induced lung injury (110), chronic obstructive diseases (111) or long-term cigarette smoking (112). In allergic animal models, airway inflammation and tracheal hyperresponsiveness are largely dependent on CD73 (113). On the other hand, in CD73 deficient mice (CD73^-/-^) sensitized with ovalbumin, there is an exacerbation of airway inflammation, as reflected by increased formation of mucus and release of pro-inflammatory mediators such like IL-1β, TNFα, IL-4 and IL-5 (114, 115). Other studies have shown that cAMP released from human CD4^+^ T lymphocytes inhibits T cell proliferation (116) and modulates differentiation of human monocytes into dendritic cells, effects that are mediated *via* A_2A_ and A_2B_ receptors (117). Likewise, in the experimental autoimmune uveitis mice model, cAMP released from T cells functions as an important source of extracellular adenosine, which in turn contributes to the immunosuppressive function of regulatory T cells (118). Finally, expression of A_2B_ receptors is increased in lungs of patients with COPD or idiopathic pulmonary fibrosis (60). The precise role of the cAMP-adenosine pathway in inflammatory pulmonary diseases is still not clear but it might function as a negative or positive feedback loop limiting or enhancing the output signal initiated by intracellular cAMP.

Currently, there are no clinical trials exploring compounds that target molecules involved in the extracellular cAMP-adenosine pathway (ABCC/MRP, ecto-phosphodiesterases and CD73) for chronic inflammatory airway diseases. However, some Phase I and II studies are testing CD73 inhibitors (e.g., HLX23, LY3475070, IPH5301, AK119, cpi-006, Sym024, IBI325, ORIC-533 and MEDI9447) alone or combined with other agents in different solid tumors, metastatic cancer and COVID-19 (NCT: 04797468, 04148937, 05143970, 04516564, 04572152, 05173792, 03454451, 04672434, 05246995, 05227144, 03381274 and 04668300) with promising results (119, 120). In addition, Phase I and II clinical trials evaluated the safety and efficacy of an MRP-1 inhibitor (sulindac) in patients with advanced melanoma (121)(EUCTR: 2006-006051-12); however, this study was terminated prematurely. Several other clinicals trials (NCR: 00430300, 01640990, 05262218, 03774290, 02635945, 01939587, 04606069) investigated the role of selective adenosine receptor agonists and antagonists (e.g., GW328267X, UK-432097, EPI-2010, CVT-6883, QAF 805, PBF-680 and Regadenoson) in various respiratory diseases, including asthma, COPD, allergic rhinitis, acute lung injury and COVID-19, however, almost all of them were discontinued due to insufficient therapeutic efficacy and/or incidence of side effects (61, 122–124). Since cAMP efflux depends on the preceding increase in intracellular cAMP, future preclinical studies and clinical trials should explore the therapeutic effects of combining treatment with bronchodilator or anti-inflammatory drugs, such as corticosteroids, β_2_-adrenoceptor agonists, or PDE inhibitors, with inhibitors of either cAMP efflux or the extracellular cAMP-adenosine pathway, to elucidate the potential therapeutic role of this biochemical pathway in inflammatory airway diseases.

## Conclusion and Future Perspectives

The discovery of cAMP by Sutherland and colleagues almost 60 years ago provided the basis for the concept of cAMP as an intracellular second messenger (125). Nevertheless, as illustrated in **Figure 1**, we are now beginning a new phase of understanding the role of cAMP as an autocrine and/or paracrine signaling molecule, in which the extracellular cAMP-adenosine pathway is able to modify cAMP signaling initiated in the intracellular environment (82).

In the airways, cAMP is the main signaling molecule implicated in bronchodilation caused by endogenous catecholamines or beta-adrenergic agents. Even after the identification of membrane transporters capable of exporting cAMP out of cells, the efflux of cAMP from airway epithelial or smooth muscle cells was considered a simple mechanism to reduce intracellular cyclic nucleotide concentrations (102). However, the discovery of an extracellular enzymatic system in airways that converts cAMP into adenosine (92) and can affect the primary cellular response initiated by intracellular cAMP (54) opened new perspectives on how to envision the role of cAMP in airway physiology, highlighting the importance of the mechanisms of cAMP extrusion and its function as an alternative source of extracellular adenosine.

As observed with adenosine, the regulatory effects of extracellular cAMP on the airways will depend on the ectoenzymes and adenosine receptor subtypes expressed in each cell, and on the amount of cAMP released into the extracellular compartment. Although we have revealed bronchoconstrictor effects of the extracellular cAMP-adenosine pathway, the relevance of the extracellular cAMP in inflammatory lung diseases is just beginning to be explored. Considering that increases in intracellular cAMP concentrations are usually followed by efflux of cAMP (88), it is important to explore the impact of the extracellular cAMP-adenosine pathway on the therapeutic effects of bronchodilators and anti-inflammatory drugs used in chronic respiratory diseases, such as glucocorticoids and classical PDE inhibitors, known to increase intracellular cAMP (126–129).

**Figure 2:**
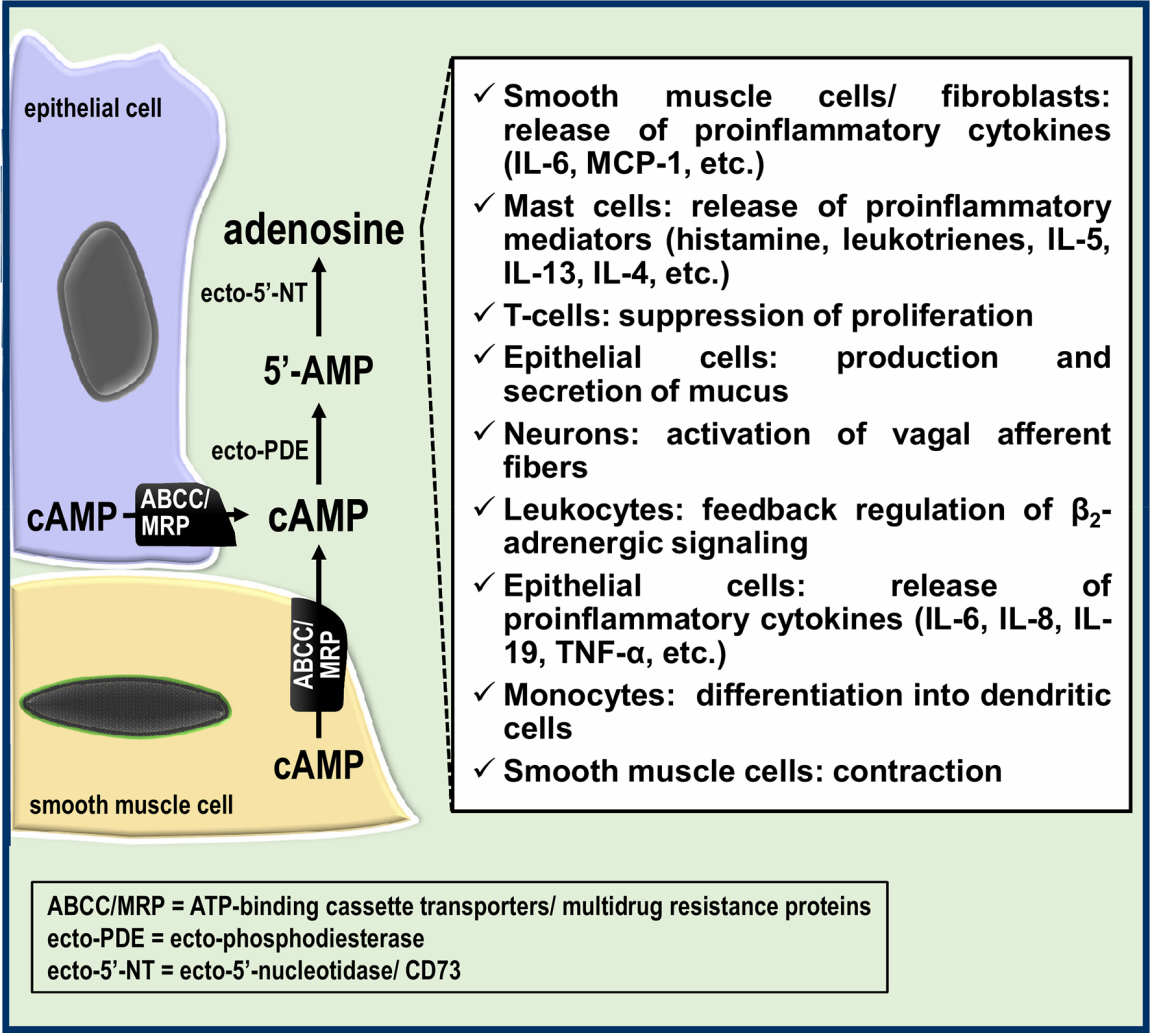
Potential roles of the extracellular cAMP-adenosine pathway in the airways. Part of the cAMP formed in epithelial and smooth muscle cells is transported by ABCC/MRP transporters to the extracellular milieu, giving rise to extracellular adenosine, which in turn can modulate the airway immune response and bronchial reactivity.

In view of the modulatory effects of adenosine on inflammatory responses and innate immune cell function, there is a potential role for cAMP efflux in airway inflammation/immune responses and bronchial reactivity (**Figure 2**). The existence of the extracellular cAMP-adenosine pathway in the airways also suggests innovative therapeutic possibilities and new pharmacological targets in airway cells, such as distinct ABCC/MRP transporters and ectoenzymes such ecto-PDEs. Future studies will be needed to decipher the precise role of the extracellular cAMP-adenosine pathway in airway inflammation and immune responses.

## Author Contributions

EP and RG contributed to conception and design of the review. RG wrote the first draft of the manuscript. EP, NS, EJ, and RG wrote the sections of the manuscript. All authors contributed to manuscript revision, read, and approved the submitted version.

## Funding

This work was supported by Fundação de Amparo à Pesquisa do Estado de São Paulo (Fapesp, grant #2018/21381-5) and financed in part by the Coordenação de Aperfeiçoamento de Pessoal de Nível Superior - Brazil (CAPES) - Finance Code 001. RG is a research fellow from Conselho Nacional de Desenvolvimento Científico e Tecnológico (CNPq; grant # 310498/2019-8. EP is a PNPD postdoctoral fellow and NS is a MSc fellow from Capes, Brazil.

## Conflict of Interest

The authors declare that the research was conducted in the absence of any commercial or financial relationships that could be construed as a potential conflict of interest.

## Publisher’s Note

All claims expressed in this article are solely those of the authors and do not necessarily represent those of their affiliated organizations, or those of the publisher, the editors and the reviewers. Any product that may be evaluated in this article, or claim that may be made by its manufacturer, is not guaranteed or endorsed by the publisher.
